# The art and science of selecting graduate students in the biomedical sciences: Performance in doctoral study of the foundational sciences

**DOI:** 10.1371/journal.pone.0193901

**Published:** 2018-04-03

**Authors:** Hee-Young Park, Oren Berkowitz, Karen Symes, Shoumita Dasgupta

**Affiliations:** 1 Department of Medical Sciences & Education, Boston University School of Medicine, Boston, Massachusetts, United States of America; 2 Department of Health Systems Management, Ariel University, Ariel, Israel; 3 Department of Biochemistry, Boston University School of Medicine, Boston, Massachusetts, United States of America; 4 Department of Medicine, Biomedical Genetics Section, Boston University School of Medicine, Boston, Massachusetts, United States of America; Medical College of Wisconsin, UNITED STATES

## Abstract

The goal of this study was to investigate associations between admissions criteria and performance in Ph.D. programs at Boston University School of Medicine. The initial phase of this project examined student performance in the classroom component of a newly established curriculum named “Foundations in Biomedical Sciences (FiBS)”. Quantitative measures including undergraduate grade point average (GPA), graduate record examination (GRE; a standardized, computer-based test) scores for the verbal (assessment of test takers’ ability to analyze, evaluate, and synthesize information and concepts provided in writing) and quantitative (assessment of test takers’ problem-solving ability) components of the examination, previous research experience, and competitiveness of previous research institution were used in the study. These criteria were compared with competencies in the program defined as students who pass the curriculum as well as students categorized as High Performers. These data indicated that there is a significant positive correlation between FiBS performance and undergraduate GPA, GRE scores, and competitiveness of undergraduate institution. No significant correlations were found between FiBS performance and research background. By taking a data-driven approach to examine admissions and performance, we hope to refine our admissions criteria to facilitate an unbiased approach to recruitment of students in the life sciences and to share our strategy to support similar goals at other institutions.

## Introduction

In selecting the next generation of biomedical scientists for our increasingly science- and technology-driven society, graduate school admissions committees around the country have the formidable task of weighing quantitative and qualitative elements of an applicant’s record in order to predict which prospective students will grow into the best scientists. Many admissions committees have attempted to take a holistic approach to this process, as has been successfully utilized in the world of medical school admissions [1]. One goal of these approaches is to increase the diversity of life science students and remain open to variety of applicants from non-traditional backgrounds and under-represented groups.

In a holistic admissions process, the process is designed to include evaluation of a variety of aspects of a student’s record by assessing a student’s academic preparation, research experience, personal qualities, and other markers of readiness for doctoral study. Notably, the identification of these other markers that ostensibly predict success in academic, research, and other domains of graduate school and beyond are ill-defined, can be based on intuition, and are inconsistently ranked in importance by scientists practicing in an academic setting [2]. Scientists have taken a particularly unscientific approach to weighing admissions criteria of prospective doctoral students, and these practices may unintentionally promote the selection of applicants like the admissions evaluators themselves rather than holistic evaluation of applicants’ skills and preparation [3]. Furthermore, as our awareness of the need for scientists in a diversity of settings [4] and career paths grows [5], we must re-evaluate whether our priorities upon admissions match the jobs for which we are preparing scientists.

Two groups recently sought to take a more measured approach towards characterizing the predictive value of different aspects of the admissions file for doctoral students at the University of California, San Francisco (UCSF) [6–7], and medical physics graduate students at Wayne State University School of Medicine [8]. The UCSF study focused on “successful” and “underperforming” students, as identified by faculty with whom they interacted during the course of their graduate studies, especially during the laboratory components of their training. This study identified the variables of subject GRE score and years of research experience prior to graduate school as significantly different between the successful and underperforming groups. However, these conclusions can only be as strong as the initial method of dividing the students into successful versus underperforming groups, which may vary significantly between institutions and even between individual admissions committee members [9]. For example, in defining success one faculty member may focus on research productivity while another may focus on ability to earn a high profile faculty position after graduation. Similarly, the Wayne State study suggested that program faculty members are good judges of students’ potential. However, the potential implicit biases of the faculty further highlight the need for each institution to take an unbiased, systematic approach towards identifying reliable predictors for success in graduate school.

Boston University School of Medicine (BUSM) recently engaged in a substantial revision of the core doctoral curriculum [10] in which the majority of PhD students in the Division of Graduate Medical Sciences (GMS) are participants. This Foundations in Biomedical Sciences (FiBS) curriculum was designed to help students develop the expected competencies of professional scientists, especially in terms of critical thinking and interdisciplinary approach to science. With our cohort of students entering this defined, uniform curriculum, we chose to explore whether there were data collected during admissions that could be associated with a student’s performance during the required formal didactic interdisciplinary scientific portion of their graduate training [10]. Because of the centralized structure of the admissions process and the core doctoral curriculum at BUSM, the school is especially well-positioned to evaluate correlations between admissions criteria and academic performance during the first year of graduate school in our student cohort.

## Materials and methods

The student cohort in this study consisted of first year doctoral students at BUSM matriculating from 2011 to 2014. The admissions process and the FiBS curriculum did not undergo any major changes during this period, and the course grading was standardized across all years of the study and across all participating programs. Course grades were primarily based on a combination of performance on take-home problem sets; in-class, timed quizzes and exams; untimed take-home exams; and small group discussions (graded on a rubric that includes active participation in the in-class paper discussion and submission of discussion questions prior to the session). These activities contributed to the assessment of doctoral program of study learning objectives (Text Box 1), particularly objectives 2–4. The students were all enrolled in Programs in the Biomedical Sciences and included Biochemistry, Biophysics, Cell and Molecular Biology, Genetics and Genomics, Immunology Training Program, Nutrition & Metabolism, Microbiology, Molecular and Translational Medicine, Oral Biology, Pathology, Pharmacology, and Physiology. Admissions data were examined for these students and included academic degrees, institutions of previous study, transcript(s), undergraduate GPA, GRE quantitative and verbal scores, employment history, publications, personal statement, and letters of reference. Analysis of these data was granted exempt status by the BUSM IRB (protocol #H-33296).

**Box 1. Learning Objectives for Doctoral Program of Study at Boston University School of Medicine**

1. Generate an original body of work in the biomedical sciences that reflects critical thinking and independent thought.
2. Generate an original body of work in the biomedical sciences that reflects critical thinking and independent thought.
3. Generate an original body of work in the biomedical sciences that reflects critical thinking and independent thought.
4. Generate an original body of work in the biomedical sciences that reflects critical thinking and independent thought.
5. Demonstrate competencies in advanced research skills and critical thinking.
6. Demonstrate competencies in advanced research skills and critical thinking.
7. Develop the ability to communicate orally and through writing within their chosen field of expertise, with specialists and non-experts.
8. Demonstrate a commitment to professional development and continued learning in their chosen field

The doctoral programs in the Division of Graduate Medical Sciences at Boston University School of Medicine are designed to train scholars to be leaders in their respective fields of biomedical research. Trainees become fluent in their areas of specialization, as well as develop competencies that provide the foundation for continued learning in their chosen field. Trainees will demonstrate and apply the professional and scientific skills necessary to benefit society.

Program failure in FiBS is defined as having FiBS GPA below 3.0. When students received a C+ (GPA 2.3) or below in individual courses, they repeated the course and their original performance still contributed to their GPA. All students, especially students experiencing academic difficulty, were offered tutoring by upperclass students through a formalized tutor program supported by the institution. High Performers were defined as those students with a total FiBS GPA equal or greater to 3.5. The competitiveness of the undergraduate institution was scored based on Barron’s Profiles of American Colleges [11] on a scale of one to seven, with seven representing the most competitive institution. Prior research experience and research environment were determined by consensus agreement between two faculty members who read independently through de-identified admissions data and rated each student on a scale of one to three with three representing the most rigorous research experience. Students matriculating from foreign institutions were excluded from part of the analysis where their institution and research background could not be objectively ranked.

Research background was rated by two independent reviewers upon evaluation of redacted application materials, most importantly the personal statement, letters of reference, and resumes of the applicants. The rubric was a three point scale (3 = substantial experiences, post graduate research or Master’s thesis; 2 = moderate experience, own project during undergraduate; 1 = introductory experience, summer research or other short experience outside of class), and ratings were achieved by consensus evaluation by authors HYP and SD. Research environment (whether the student’s undergraduate institution or elsewhere) was categorized using a systematic rubric as well (1 = liberal arts college; 2 = biotech/pharmaceutical industry; 3 = research institution/university).

Descriptive statistics, such as mean with standard deviation and proportions, were calculated for admission variables. Ordinal logistic regression models were built to calculate the effect of admissions variables on FiBS performance. Overall GPA at the end of the FiBS curriculum was used as the dependent variable and ranked at three levels: Fail (GPA < 3.0), Pass (3.5 > GPA > 3.0), and High performer (GPA ≥ 3.5). Both crude and adjusted odds ratios (OR) with 95% confidence intervals (95% CI) were calculated for each of the admissions variables. Test of Parallel Lines was performed to verify the assumption of proportional odds for ordinal regression. Data analysis was done in PASW Version 18 (IBM Chicago, IL 2009).

## Results

A total of 95 students matriculated to the FiBS curriculum from 2011 to 2014. S1 File shows data gathered for the study. The average age was 27.5 (SD = 3.1), 66% identified as female, and 76% came from US colleges and universities. The majority of the students identified as White (49%), followed by 9% Asian, 6% Hispanic/Latina/o and 4% Black/African-American, and 32% chose not to report their race/ethnicity. The competitiveness of undergraduate institutions averaged at 5.4 (SD = 2) on a scale of one to seven, with seven being competitive. The average undergraduate GPA was 3.43 (SD = 0.36) and GRE scores averaged at the 74^th^ and 76^th^ percentiles for the quantitative and verbal scores, respectively. A total of 83 (87%) students had complete data for analysis of their research background as shown in S1 File. Students had strong research backgrounds overall with an average of one publication per student. Students had highly rated prior research experiences and environments at about 2.5 on a scale of one to three (Table 1), with three being the most highly rated.

**Table 1:** Demographic profile for graduate students enrolled in FiBS curriculum.

	Overall (n = 95)		Fail (GPA < 3.0, n = 17)		Pass (3.5 > GPA ≥ 3.0, n = 41)		High Performer (GPA ≥ 3.5, n = 37)	
Variables	Mean	SD	Mean	SD	Mean	SD	Mean	SD
FiBS GPA	3.35	0.36	2.82	0.09	3.24	0.14	3.72	0.16
Age	27.5	3.1	26.9	2.7	27.8	3.6	27.4	2.7
Female (n, %)	63	66%	16	94%	22	54%	25	68%
USA applicant (n, %)	72	76%	13	77%	34	83%	25	68%
Competitiveness of undergrad (scale of 1–7)^1^	5.4	2	3.7	2.4	5.3	1.9	6	1.5
Undergrad GPA	3.43	0.36	3.29	0.35	3.36	0.37	3.58	0.30
GRE quantitative %ile	73.7	16.1	63	20.4	73.5	15.2	79	12.3
GRE verbal %ile	75.5	18.8	69.2	20.4	74.1	19.1	79.8	17.2
#Publications	1.1	3.2	1.2	1.7	0.6	1	1.5	4.8
Research experience (scale of 1–3)^2^	2.5	0.6	2.6	0.5	2.5	0.6	2.4	0.6
Research Environment (scale of 1–3)^3^	2.6	0.7	2.5	0.9	2.7	0.7	2.6	0.7

^1^Competitiveness of Undergrad was rated by investigators on ascending Likert scale based on Barron’s Profiles of American Colleges, with 7 corresponding to the most competitive institutions.

^2^Research Experience.

^3^Research Experience was rated by investigators on ascending Likert scale based on personal statement, letters of reference, and resumes, with three corresponding to the most rigorous research experiences. These variables were able to be categorized for 83 students in the overall cohort.

There were 78 (82%) students who passed the FiBS curriculum with a GPA ≥ 3.0 (Pass and High Performer groups combined). Seventeen students (18%) failed (GPA < 3.0). The highest performers passed the FiBS curriculum with a GPA ≥ 3.5. There were 37 (39%) students in this category and they had an average GPA of 3.72 (SD = 0.16) (Table 1).

### Ordinal regression analysis

Ordinal regression analysis enabled us to estimate the likelihood of progressing from each category (Fail/Pass/High Performer) based on each incremental increase of the independent variable. Three factors significantly contributed to better FiBS performance in the adjusted multivariate model. Students who came from more competitive undergraduate institutions performed better (aOR 1.76, 95%CI: 1.21–2.58), as did students with stronger undergraduate GPA (aOR 8.96, 95%CI: 1.37–58.79). Age also became a significant factor in the adjusted model (aOR 1.26, 95%CI: 1.04–1.52). GRE scores were mildly contributory in univariate analysis but did not remain significant in the adjusted model (Quantitative GRE: crude OR 1.04, 95%Cl: 1.02–1.07; Verbal GRE crude OR 1.02, 95%Cl: 1.00–1.04). The number of publications, research experience, and research environment did not demonstrate any significant contributions to improved FiBS performance (Table 2). Gender was not found to be statistically significant. After adjusting for all independent variables, female gender did have a high degree of association with improved performance, but this did not reach statistical significance (aOR 2.27, 95% CI: 0.59–8.75). In addition, whether the student attended a US or foreign institution did not correlate with a significantly improved FiBS performance.

**Table 2:** Ordinal logistic regression models for improved performance in FiBS curriculum: Crude and adjusted values. Dependent variable ranked as: Fail/Pass/High performer, as previously defined. All variables included in adjusted model. Test of Parallel Lines = 0.57 for model. Significant correlations are indicated in bold.

	Crude values				95% CI		Adjusted values				95% CI	
Variables	Estimate	Std. Error	Sig.	OR	Lower	Upper	Estimate	Std. Error	Sig.	aOR	Lower	Upper
Age	0.01	0.06	0.82	1.01	0.90	1.14	0.23	0.10	0.02	**1.26**	1.04	1.52
Female	-0.40	0.41	0.34	0.67	0.30	1.51	0.82	0.69	0.23	2.27	0.59	8.75
USA applicant	-0.55	0.46	0.23	0.58	0.24	1.42	-1.45	1.37	0.29	0.23	0.02	3.43
Competitiveness of undergrad	0.42	0.13	0.00	**1.52**	1.17	1.96	0.57	0.19	0.00	**1.76**	1.21	2.58
Undergrad GPA	1.80	0.61	0.00	**6.04**	1.81	20.13	2.19	0.96	0.02	**8.96**	1.37	58.79
GRE quantitative %ile	0.04	0.01	0.00	**1.04**	1.02	1.07	0.04	0.03	0.11	1.04	0.99	1.10
GRE verbal %ile	0.02	0.01	0.05	**1.02**	1.00	1.04	0.03	0.02	0.17	1.03	0.99	1.08
#Publications	0.06	0.08	0.42	1.06	0.92	1.23	-0.02	0.10	0.84	0.98	0.81	1.18
Research experience	-0.43	0.35	0.22	0.65	0.33	1.29	0.13	0.57	0.82	1.14	0.37	3.46
Research Environment	-0.02	0.29	0.94	0.98	0.56	1.71	0.18	0.44	0.69	1.19	0.50	2.82

## Discussion

This study has allowed us to investigate predictors of academic success during the formal didactic portion of graduate training at BUSM. Interestingly, we have found items associated with improved performance in this initial phase of training. When assessing basic proficiency required to pass the first year of study, the most impactful criteria were the students’ undergraduate GPA and the competitiveness of their undergraduate institution. Age also became a positive factor in the adjusted analysis. While gender did demonstrate a high degree of association with improved performance among female students, this finding did not reach statistical significance, perhaps because of a lack of statistical power from a limited sample size or simply a random association. Identifying factors associated with both being a higher performer and being able to pass are equally important. The goal is to select both the high performers and those who are able to pass the didactic aspect of their training. This approach takes into account that some applicants may come from a non-traditional background, which can be associated with lower standardized test scores, lower GPA, and less competitive institutions.

In the case of non-traditional students, it is important for the school to be able to identify trainees from a diversity of backgrounds who will be able to bring unique qualities to their respective fields, while still achieving proficiency in the classroom. Notably, for female students and students from underrepresented backgrounds, additional barriers are present that begin to dissuade them from pursuing careers in research as early as during graduate school [12, 13]. Awareness of these barriers are important in supporting students’ progress through both the didactic and research phases of their graduate training.

By preferentially selecting applicants with the highest GREs, GPAs, competitive undergraduate institutions, and extensive research experiences, an opportunity may be missed in training future scientists who are committed to contributing in settings both inside and outside of academia, to understanding health-care disparities at a basic level, to engaging in development of policy and advocacy for basic science, and to participating in any number of other critical activities where scientific perspective is needed. Institutions should avoid this conflict by exploring their admissions processes in a data driven way, as outlined in this study, in order to understand which criteria make sense for their curriculum, their metrics of success, and their overall institutional mission. For similar reasons, a minimum threshold approach to medical school admissions has been proposed, where above the numerical cut-offs, non-academic considerations could be introduced into the admissions process [14]. Notably, proceeding in a minimum threshold direction in terms of admissions may have consequences for an institution’s ranking by various agencies and publications, and therefore before a debate about the validity of these strategies can be initiated, a more nuanced understanding of what admissions data is actually indicating is needed.

The criteria identified by this study as being associated with academic performance during the first year of graduate school are, in many ways, what you would expect to find when measuring success in terms of classroom-based activities, but exploring these data allow us to develop evidence-based guidelines to inform our admissions process. For example, the fact that students coming from more competitive undergraduate settings are more likely to pass suggests that students from less competitive settings may benefit from closer examination of their GPA as a counterbalance and potentially from pre-matriculation interventions such as preparatory coursework. The lack of correlation between prior research experience and academic success in our analysis might be an artifact of a selection bias where we have mostly chosen students with high overall levels of research experience, or it might reflect the idea that research experience is not especially helpful in the classroom phase of their education. The virtues of prior research experience may be useful in the later research-intensive phases of the PhD program.

As new classes of graduate students are admitted and these cohorts progress through graduate school, the intention is to continue tracking students from admission to graduation and beyond to assess successful completion of the qualifying exam, selection for trainee fellowships, number of conference presentations and publications over time, completion of and time to degree, as well as time to employment post-graduation. In the meantime, the goal is to continue to systematically review the criteria available to make admissions decisions and to continuously revisit how we characterize a successful scientist using the broad criteria defined above, so as to capture the critical contributions of scientists in a diverse set of venues. Our initial analysis has been limited to the didactic portion of the curriculum but in order to continue to build a diverse biomedical workforce, we must be open to a broader definition of success that is not limited to the classroom portion of graduate training. As we refine our approach to determining which admissions criteria are meaningful, we will also begin to look at whether our efforts have increased the diversity of our students.

Importantly, GRE scores only weakly contributed to academic success in the first year of graduate school in univariate analysis but did not remain significant in the adjusted model. This finding has also been demonstrated in doctoral programs in the biological sciences at Vanderbilt University [15], and the ability of the GRE to predict other aspects of a productive graduate career has also been called into question [15, 16]. In this context it is critical to acknowledge that GRE scores have also been associated with standardized test disparities along demographic lines including gender, ethnicity, and socioeconomic status [17]. Taken together, the graduate education community in the biological sciences has begun seriously considering elimination or de-emphasis of the GRE in admissions decisions [18]. By exploring our data, we can develop guidelines to counterbalance potential amplification of disparities via emphasis on standardized tests by generating a strategy to systematically evaluate other admissions criteria. For example, as multiple choice questions were not an assessment method used in the Boston University FiBS courses, we expected minimal impact of biases contributing to GRE performance on FiBS performance, and in fact identified stronger predictive correlations with undergraduate GPA, and to a lesser extent, competitiveness of undergraduate institution and age. Similarly, a recent study from the University of Puerto Rico, where students typically score below the 15^th^ percentile on the GRE, indicated that a composite score that factors together quantitative academic elements and criteria such as years of research experience and publication record is used for admissions criteria [16]. This approach gives the admissions committee freedom to exercise judgment on several components of an applicant’s record. Furthermore, de-emphasis of scores alone has not been shown to sufficiently improve the recruitment of under-represented groups in science [17]. We aim to explore this approach for more diversified applicants with the goal of improving the representation and retention of groups underrepresented in science.

Our ultimate goal is to create a data-driven assessment rubric that will allow an admissions committee to make informed decisions about the likelihood of a given applicant’s success in graduate school given their particular qualifications and the range of outcomes delineated above. These efforts must be paired with deliberate recruitment efforts to encourage applications from students with a wide range of demographic characteristics and qualifications. Ideally, these pre-matriculation assessments will also help to identify areas where a student may require additional assistance in order to successfully achieve milestones in graduate education, particularly in the case where other holistic elements of their application suggest the greater good of offering them a seat in the graduate program. The evidence-based assessment of prospective students is a critically important element of the unbiased evaluation of an applicant pool. The goal of this approach is to ultimately facilitate the assembly of a diverse biomedical workforce to bring the skills of professional scientists to bear in an increasingly wide range of arenas in modern society.

## Supporting information

S1 File Primary data. (XLSX)

## Abbreviations

BUSM: Boston University School of Medicine
FiBS: Foundations in Biomedical Sciences
GMS: Graduate Medical Sciences
GPA: grade point average
GRE: graduate record examination
UCSF: University of California, San Francisco
